# Stress Mediators and Immune Dysfunction in Patients with Acute Cerebrovascular Diseases

**DOI:** 10.1371/journal.pone.0074839

**Published:** 2013-09-19

**Authors:** Arthur Liesz, Holger Rüger, Jan Purrucker, Markus Zorn, Alexander Dalpke, Markus Möhlenbruch, Stefan Englert, Peter P. Nawroth, Roland Veltkamp

**Affiliations:** 1 Department of Neurology, University Heidelberg, Heidelberg, Germany; 2 Department of Internal Medicine, University Heidelberg, Heidelberg, Germany; 3 Department of Infectious Diseases, Medical Microbiology and Hygiene, University Heidelberg, Heidelberg, Germany; 4 Department of Neuroradiology, University Heidelberg, Heidelberg, Germany; 5 Institute of Medical Biometry and Informatics, University Heidelberg, Heidelberg, Germany; National University of Singapore, Singapore

## Abstract

**Background:**

Post-stroke immune depression contributes to the development of infections which are major complications after stroke. Previous experimental and clinical studies suggested that humoral stress mediators induce immune dysfunction. However, prospective clinical studies testing this concept are missing and no data exists for other cerebrovascular diseases including intracerebral hemorrhage (ICH) and TIA.

**Methods:**

We performed a prospective clinical study investigating 166 patients with TIA, ischemic and hemorrhagic stroke. We measured a broad panel of stress mediators, leukocyte subpopulations, cytokines and infection markers from hospital admission to day 7 and on follow-up after 2–3 months. Multivariate regression analyses detected independent predictors of immune dysfunction and bacterial infections. ROC curves were used to test the diagnostic value of these parameters.

**Results:**

Only severe ischemic strokes and ICH increased some catecholamine metabolites, ACTH and cortisol levels. Immunodysfunction was eminent already on hospital admission after large brain lesions with lymphocytopenia as a key feature. None of the stress mediators was an independent predictor of lymphocytopenia or infections. However, lymphocytopenia on hospital admission was detected as an independent explanatory variable of later infections. NIHSSS and lymphocytopenia on admission were excellent predictors of infection.

**Conclusions:**

Our results question the present pathophysiological concept of stress-hormone mediated immunodysfunction after stroke. Early lymphocytopenia was identified as an early independent predictor of post-stroke infections. Absence of lymphocytopenia may serve as a negative predictive marker for stratification for early antibiotic treatment.

## Introduction

Infectious complications are a leading cause of morbidity and mortality after stroke [1], [2]. The most common infections are pneumonia and urinary tract infections. Nearly all pneumonias occur within the first week after stroke [3]. Besides well-known risk factors for pneumonia including stroke severity, age and dysphagia [4] a stroke-induced immunosuppression syndrome (SIDS) has been identified. Lymphocytopenia and reduced lymphocyte and monocyte function are the hallmarks of this syndrome [5]–[9]. SIDS has been associated with a substantially increased susceptibility to bacterial infections in experimental stroke models and stroke patients [10], [11]. These effects depend on lesion size and were observed mainly after large ischemic stroke [12], [13].

Already early clinical observations described the influence of brain ischemia on peripheral immune cells and proposed the sympathetic nervous system as the mediator of brain-immune interaction^14, 15^. Indeed, an increase of stress mediators including catecholamines and cortisol can be detected in stroke patients with extensive brain lesions [6], [14], [15]. Also, pharmacological blockade of beta-adrenergic receptors in experimental ischemia studies resulted in a partial normalization of immunological impairment [8]. These results led to the current concept that acute brain lesions result in an increased release of catecholamines and corticosteroids which downregulate the peripheral immune system and increase the susceptibility to infections [7], [10], [11], [16].

A detailed review of the data underlying this concept reveals important limitations. Stress mediators have not been adequately established as independent predictors of post-stroke infections in clinical studies. Evidence for a causal relationship between the humoral stress response and immunomodulation in stroke comes from a single experimental study [8] using pharmacological blockade of catecholamine and glucocorticoid receptors only. Moreover, the concept has not been examined in other common cerebrovascular diseases such as transient ischemic attacks (TIA) and intracerebral hemorrhage (ICH) which also induce peripheral immune changes [17].

We conducted a prospective study investigating the correlation and independent predictors of stress mediators, cellular and humoral immune markers and infectious complications in patients with TIA, ischemic stroke, and ICH compared to matched controls.

## Methods

The study was designed as a prospective, single center observational study conducted from 10/2011 until 11/2012 and was approved by the local ethics committee of the University Heidelberg (S-348/2011). All procedures were performed after written informed consent was obtained by the patient or a legal representative. Inclusion criteria were study enrollment within 24 h after symptom-onset resulting from a transient ischemic attack (TIA), ischemic stroke or intracerebral hemorrhage (ICH). Exclusion criteria were recent clinical infection (fever, cough, antibiotic treatment, laboratory markers) within 14d prior to admission, a chronic immunological disorder, preexisting immunosuppression, cancer and pregnancy.

For the control group, patients were included that presented at the Department of Ophthalmology for pre-surgical screening for cataract operation. Patients were selected to be in the same range for age and gender of patients with cerebrovascular disorders. Clinical data and blood markers were assessed for control patients by the same procedures as for patients with cerebrovascular disorders except for 24-hour urine collection. Blood was obtained only once in control patients.

Patients received routine cranial imaging by CT (Somatom Sensation 16) and/or 3 T MRI (Trio and/or Verio; all Siemens Medical Systems) to detect ischemic or hemorrhagic brain lesions. In TIA patients cerebral infarction was ruled out by MRI. The earliest image revealing the diagnosis was used to assess ischemic lesion or hematoma volume, when repeated images where performed, the one with the largest lesion demarcation was used for volumetry. Volume was calculated by integrating areas on each image multiplied by section diameter using a Syngo Multimodality workplace with syngoMMWP-software (version VE31A, Siemens).

Patients with cerebrovascular diseases underwent blood sampling on admission (day 1), days 2, 3 and 7, and at follow-up at 60–90 days later. We measured serum catecholamines and metabolites, ACTH, cortisol, differential blood cell count, cytokines, CRP and high sensitivity procalcitonin (hsPCT) in the central laboratory of the University Hospital Heidelberg by the same procedures used and validated for routine diagnostic analysis. Differential blood cell counts were obtained by automated flow cytometric analysis. Cortisol and ACTH concentrations were measured by chemiluminescence immunoassays (CLIA), all plasma catecholamines were analyzed by high-performance lipid chromatography (HPLC) and serum metanephrin/normetanephrin were detected by enzyme-linked immunoassays (ELISA). CRP was analyzed by turbidimetry. Assays for hsPCT were measured by electrochemoluminescence immunoassay (ECLIA). Normal value limits of blood biomarkers according to the manufacturers were as follows: cortisol 50 ng/ml, ACTH <46 pg/ml, adrenalin <464 pmol/l, noradrenalin <1625 pmol/l, dopamine <560 pmol/l, metanephrin <90 pg/ml, normetanephrin <180 pg/ml, CRP<5 mg/l, hsPCT <0.05 ng/ml.

Routine microbiological analyses, chest x-ray and further examinations related to infectious complications were documented for the first 7d. In case of clinical and/or laboratory signs of infection, all patients received aerobic and anaerobic cultures of blood, sputum, tracheal aspirate, bronchoalveolar lavage, cerebrospinal fluid (CSF) or urine, respectively, at the treating physician’s discretion. Positive bacterial infection was scored only in case of positive microbiological analysis, indicated by one of the following: presence of typical pathogenic bacteria, significant numbers of colony-forming units (CFU) for facultative pathogenic bacteria excluding sample contamination or any positive bacterial culture of physiologically sterile material (catheter urine sample, CSF and bronchoalveolar lavage).

Blood samples for cytokine measurement were immediately processed for plasma separation and stored at −80°C until analysis. Plasma cytokine concentrations were measured using commercial ELISA kits following the manufacturer’s protocols (R&D Systems).

Urine catecholamine concentrations (noradrenalin, metanephrin, normetanephrin and vanillylmandelic acid) were measured by HPLC of 24-hour collection urine started immediately after study inclusion. Obtained concentrations were normalized to the produced urine volume of the 24 h collection period.

### Statistical Analysis

Descriptive statistics and statistical hypothesis tests were performed using Graphpad Prism software (version 6.0b). P values <0.05 were considered to be statistically significant. Regression analyses and receiver operating characteristic (ROC) curves were calculated using IBM SPSS software (version 21). Binary logistic regression was applied for univariate analysis. Explanatory variables of the univariate analysis with a p value <0.1 for the respective dependent variable were included for multivariate analysis. Multivariate binary regression analyses were performed using a conditional forward approach by likelihood ratio. Criteria for stepwise analysis were set at 0.05 for inclusion and at 0.1 for exclusion from the model. ROC curves for the combined variables (lymphocytes×NIHSS) were plotted using predicted probabilities of combined predictors from univariate logistic regression models.

## Results

### Patient Characteristics

In total, 166 subjects were enrolled into the study. 23 patients had a transient ischemic attack (TIA), 70 patients an ischemic stroke and 43 patients an intracerebral hemorrhage. 30 patients with no neurological or immunological disorder were included as control (**Table 1**). The mean time between symptom-onset and study inclusion (d1) was 11.9 h and did not differ among groups. Approx. 78% of surviving patients could be examined for follow-up at day 60–90. Baseline variables did not differ between the appropriate groups except for atrial fibrillation that was significantly more frequent in the ischemic stroke group (**Table 1**). According to the TOAST criteria [18], stroke etiology in the ischemic stroke group was large-artery atherosclerosis in 13%, cardioembolism in 34%, small-artery occlusion in 12%, other in 2% and undetermined in 39%.

**Table 1:** Clinical characteristics of patients used for analysis in the control, transient ischemic attack (TIA), ischemic stroke (stroke) and intracerebral hemorrhage (ICH) group.

	Control	TIA	Stroke	ICH	p value
*Clinical characteristics*					
n (total: 166)	30	23	70	43	
Age (years)	70.7±8.8	63.0±10.9	69.1±10.6	66.8±14.3	0.096
Gender (male/female)	15/15	17/6	41/29	25/18	0.371
Lesion Volume (ml)	0	0	90±122	33±33	0.577#
Median lesion volume (ml)	n.a.	n.a.	21.6	20.4	
Laterality (right/left)	n.a.	11/12	31/39	25/18	0.356†
NIHSSS at admission	0	0.2±0.5	12.2±7.9	16.6±11.8	0.134#
Symptom to d1 time (h)	n.a.	12.3±7.6	12.9±7.9	10.1±7.5	0.169
Catecholamine treatment	n.a.	0	13 (19%)	19 (44%)	*0.003* ‡
Mortality (on day 7)	n.a.	0	5 (7%)	1 (2%)	0.254†
Mortality (on follow up)	n.a.	0	11 (16%)	6 (14%)	0.129†
Follow up (survivors	n.a.	17 (74%)	48 (81%)	28 (76%)	0.694†
*Pre-existing condition at admission*					
Dyslipidemia	9 (30%)	5 (22%)	19 (27%)	8 (19%)	0.649
Arterial hypertension	26 (87%)	17 (74%)	44 (63%)	27 (63%)	0.086
Diabetes	9 (30%)	4 (17%)	23 (33%)	6 (14%)	0.095
Smoking	2 (7%)	5 (22%)	15 (21%)	8 (19%)	0.336
Prev. cardiovasc. event^1^	2 (7%)	7 (30%)	22 (31%)	11 (26%)	0.065
Atrial fibrillation	2 (7%)	3 (13%)	21 (30%)	5 (12%)	*0.014*
*Premedication at admission*					
β-AR blocker	12 (4%)	7 (30%)	27 (39%)	12 (28%)	0.597
Oral anticoagulants^2^	7 (23%)	1 (4%)	13 (18%)	7 (16%)	0.305
Antiplatelet drug^3^	8 (27%)	11 (48%)	17 (24%)	11 (26%)	0.167
Sympathicoactive drugs^4^	4 (13%)	1 (4%)	4 (6%)	2 (5%)	0.434

1 previous TIA, stroke or myocardial infarction;

2 phenprocoumon, rivaroxaban;

3 aspirin, aspirin+dipyridamole, clopidogrel;

4 clonidin, oxybutinin, inhalable β2-AR agonists, doxazosin, terazosin.

Metric data is shown as mean±SD, non-metric values as absolute numbers and percentage.

# U test for stroke and ICH;

† chi square test for TIA, stroke and ICH;

‡ chi square test for stroke and ICH.

### Humoral Stress Mediators after Acute Cerebrovascular Events

Previous results [12], [13], [19] indicated an important role of lesion volume on effect size of systemic immune alterations. We therefore dichotomized patients with ischemic stroke and intracerebral hemorrhage to either small or large lesion volumes. Because a defined and validated lesion volume for discrimination does not exist, we used the median of lesion volume in the respective group (ischemic stroke: 20.8 mm^3^, ICH: 21.6 mm^3^) to dichotomize between small and large lesions.

**Figure 1:**
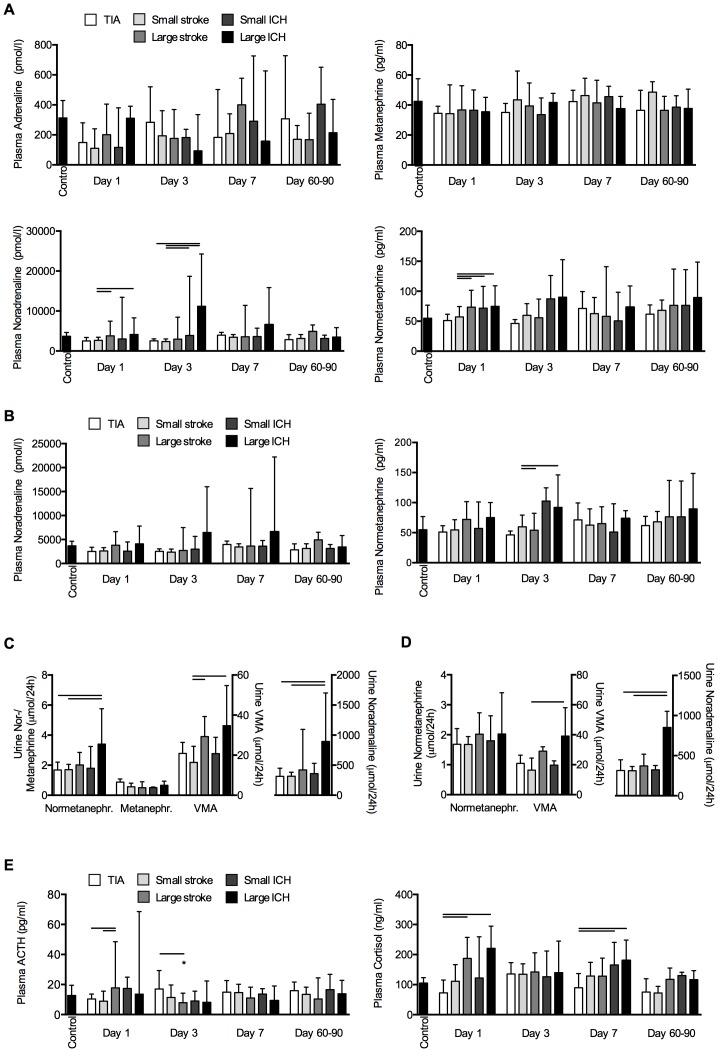
Humoral stress response in patients with acute cerebrovascular diseases. Blood plasma concentrations of indicated catecholamines and metanephrines were analyzed in (**A**) all patients (n = 166) or (**B**) only in patients not receiving catecholamine treatment (n = 134). Urin excretion of noradrenalin, metanephrines and vanillylmandelic acid (VMA) within the first 24 h after admission was investigated (**C**) in all patients with cerebrovascular events (left) or after exclusion of patients receiving catecholamine treatment during the urine collection period (right). (**D**) To investigate the HPA-axis, blood plasma concentrations of ACTH and cortisol were measured at the indicated time points after cerebrovascular events and in control patients. Group differences for all parameters were analyzed by Kruskal-Wallis H-test and Dunn’s post hoc test. Data is presented as median and interquartile range. * indicates significant (p<0.05) difference of the indicated group compared to the control group; horizontal bars indicate significant differences among groups at the respective time point.

Interestingly, we did not observe a significant difference of plasma adrenalin and metanephrin levels in any group and at any time point compared to control (**Fig. 1A**). Also serum dopamine levels were not consistently altered (**Fig. S1)**. In contrast, plasma noradrenalin concentration was significantly increased at admission and 3d after large stroke and ICH compared to small strokes. Normetanephrin concentrations were significantly increased after large stroke and in both ICH groups compared to small strokes (**Fig. 1A**). However, a significant proportion of patients with large stroke and ICH received intensive medical care including catecholamine (mainly norepinephrine) treatment (compare to Table 1). After exclusion of patients receiving catecholamine treatment no significant difference for plasma noradrenalin concentrations was detectable and a significant increase of plasma normetanephrin only remained for large ICH compared to TIA at 3d after onset (**Fig. 1B**). We investigated catecholamine concentration in 24-hour collection urine to validate the results for plasma catecholamine levels. We detected a significant increase of urine noradrenalin and normetanephrin concentration after large ICH and vanillylmandelic acid (VMA) additionally after large stroke (**Fig. 1C**). Corresponding to plasma catecholamine concentrations, exclusion of catecholamine-treated patients resulted in attenuation of this effect (i.e. only large ICH increased noradrenalin and VMA levels significantly) (**Fig. 1C**).

We additionally investigated the hypothalamic-pituitary-adrenal (HPA) axis by analysis of ACTH and cortisol levels. We detected an early significant increase of plasma ACTH and cortisol concentration after large ischemic stroke and ICH (**Fig. 1D**).

### Ischemic and Hemorrhagic Brain Lesions Induce Profound Immune Alterations

**Figure 2:**
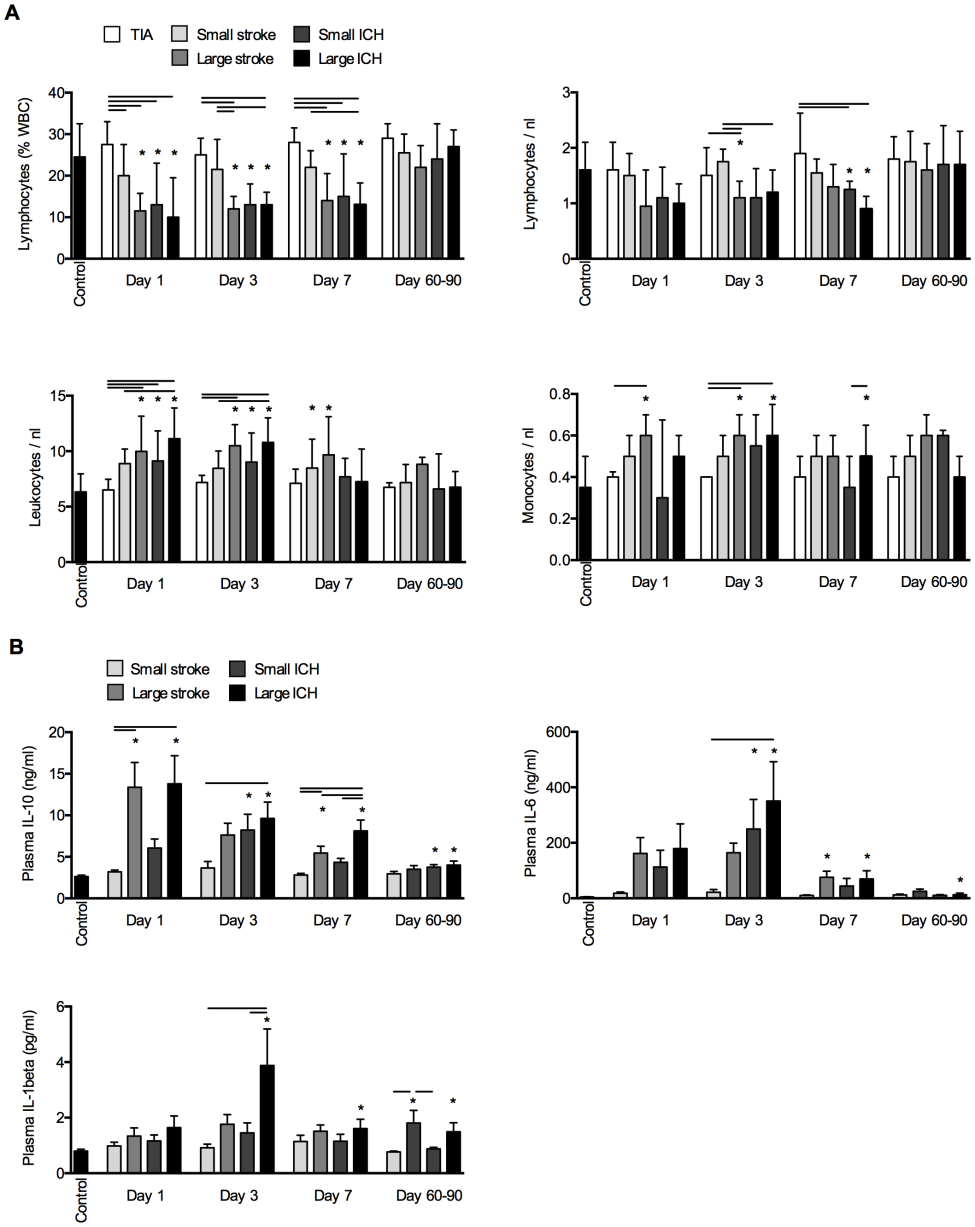
Alterations of the immune system after cerebrovascular events. (**A**) Changes of the cellular immune system were measured by the relative lymphocyte fraction, absolute lymphocyte counts, total leukocyte number and the absolute monocyte cell counts in control patients and at the indicated time points after cerebrovascular events. (**B**) Blood plasma concentrations of IL-10, IL-6 and IL-1β were determined to analyze humoral mediators of immune function. Blood cell counts and cytokines were analyzed by analysis of variance (ANOVA) after testing for normal distribution. Data are presented as mean+/− standard deviation. * indicates a significant (p<0.05) difference of the indicated group compared to control; horizontal bars indicate significant group differences at the respective time point.

We analyzed differential blood cell counts and cytokine concentrations of patients suffering from cerebrovascular diseases and controls to characterize peripheral immune function. We detected a significant reduction of the lymphocyte proportion by >60% in patients with large ischemic strokes and both ICH groups compared to TIA patients and controls already on d1. This pattern of profound lymphocytopenia after severe brain lesion persisted throughout the first week after the event (**Fig. 2A**). Also absolute lymphocyte cell counts were significantly reduced, however, with an higher variability compared to the relative lymphocyte measure. In contrast, total leukocytes and monocyte blood cell counts were significantly increased after large brain lesions from day 1 through day 7. We further characterized humoral immune function by measurement of the anti-inflammatory cytokine IL-10 and the early pro-inflammatory cytokines IL-6 and IL-1β. We detected a strong increase in plasma IL-10 concentrations already on admission (day 1) in patients having large lesions (**Fig. 2B**). Plasma concentrations for the pro-inflammatory cytokines IL-6 and IL-1β peaked at 3d after symptom-onset and showed a most pronounced increase after large ICH. Interestingly, large stroke and ICH resulted in a prolonged increase of IL-6 and IL-1β concentration until follow-up, resulting in a still 2-fold increase of IL-1β compared to controls.

### Infection Surrogate Markers and Bacterial Culture Results

**Figure 3:**
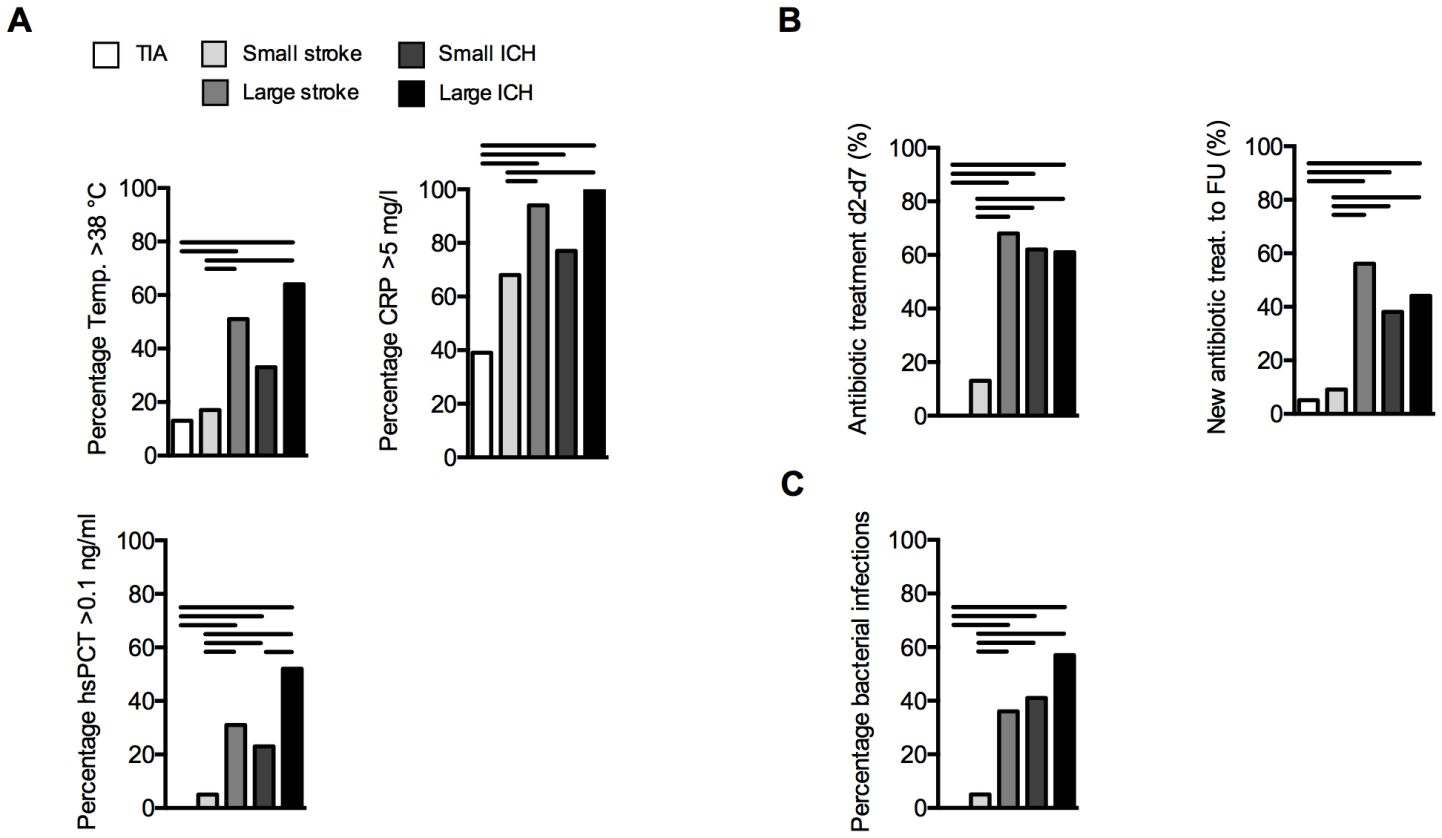
Infection markers and microbiological culture results. (**A**) The proportion of the positive infection markers febrile body temperature (>38°C), CRP>5 mg/l and hsPCT >0.1 ng/ml within the study cohorts is presented as percentage of the respective group. (**B**) The proportion of patients receiving antibiotic treatment is depicted for the first week after symptom onset (d2–d7, left panel) or for a new antibiotic treatment after hospital discharge until follow-up (FU) after 60–90d (right panel). (**C**) Bars depict the percentage of patients of the respective group with at least one pathological bacterial culture result during the first week after symptom onset (d2–d7) (cp. methods for definition). Data was tested using chi-squared tests and is presented as proportions per group. Horizontal lines indicate significant (p<0.05) group differences (chi-squared test).

Analyses of surrogate markers (fever, c-reactive protein and procalcitonin levels) for bacterial infection within the first week after symptom-onset revealed a high number of pathological results (**Fig. 3A**). A significantly higher proportion of patients with large ischemic stroke or ICH had an increase in infection markers compared to TIA and/or small strokes, respectively. Up to 60% of patients had fever and nearly 100% of large ischemic and hemorrhagic stroke patients had pathological serum CRP values >5 mg/l. In contrast, a pathological test result for hsPCT (>0.1 ng/ml) was detected in considerably fewer patients (**Fig. 3A**). Approx. 60% of patients within the large stroke or either ICH group received antibiotic treatment during the first week after onset. Moreover, a significantly higher proportion of these patient groups also received a second, independent course of antibiotic treatment after discharge from hospital (**Fig. 3B**). Large stroke and both ICH groups also resulted in significantly more positive bacterial culture results than in patients with TIA or small ischemic lesions (**Fig. 3C**). Remarkably, the proportion of patients with positive bacterial cultures was distinctly lower than indicated by the most widely used infection markers fever and CRP and also considerably lower than in patients receiving antibiotic treatment (compare to Fig. 3A,B).

### Independent Predictors of Lymphocytopenia and Bacterial Infections

To detect independent predictors of the key feature of post-stroke immunosuppression – lymphocytopenia and infection – we performed univariate regression analyses and included explanatory variables with a p value <0.1 into a multivariate regression model. The cutoff value of <13% for relative lymphocytopenia was determined using ROC curve analysis (see below). Logistic regression analysis for the dependent variable lymphocytopenia<13% on day 1 (admission) included only predictive variables on admission (**Table 2** **)**. Interestingly, among stress markers only cortisol and noradrenalin levels in all patients had a significant p value (<0.1) while catecholamine concentrations in patients without catecholamine treatment (n = 109) were not significantly correlated with lymphocytopenia. In the univariate logistic regression model for bacterial infections within the first week after admission all values obtained during the first 7d were included (**Table 3**). Markers of disease severity (NIHSSS, lesion volume, catecholamine treatment, mechanical ventilation) were all significantly correlated with development of bacterial infections. Lymphocytopenia, IL-10 and IL-6 concentrations on day 1 as well as noradrenalin and normetanephrin levels were highly correlated to the subsequent development of bacterial infections (**Table 3**).

**Table 2:** Results of univariate binary logistic regression analysis for lymphocytes <13% on day 1.

		B	OR (95% CI)	Std.OR	p value
Gender (male)		0.001	1.001 (0.499–2.006)		0.998
Age		0.033	1.033 (1.002–1.065)	1.489	*0.037*
NIHSSS		0.117	1.124 (1.075–1.176)	3.333	*<0.001*
Volume		0.008	1.008 (1.003–1.014)	2.164	*0.001*
*Medical condition and premedication*					
Diabetes		−0.402	0.669 (0.283–1.582)		0.360
PrevCardioVasc		−0.590	0.554 (0.255–1.204)		0.136
β-AR blocker		−0.149	0.861 (0.416–1.784)		0.687
Smoking		−1.025	0.395 (0.141–0.914)		*0.032*
*Blood biomarkers ad admission (d1)*					
Monocytes	0.820		2.270 (0.419–12.282)	1.198	0.341
Catecholaminetreatment d1	1.199		3.317 (1.363–8.070)		*0.008*
Noradrenalin	0.000		1.000 (1.000–1.000)	1.000	*0.024*
Normetanephrin	0.005		1.005 (0.998–1.012)	1.286	0.140
Noradrenalin(w/o Norepi.)	0.000		1.000 (1.000–1.000)	1.000	0.896
Normetanephrin(w/o Norepi.)	0.002		1.002 (0.991–1.013)	1.072	0.723
ACTH	0.004		1.004 (0.999–1.009)	1.436	0.116
Cortisol	0.003		1.003 (1.000–1.006)	1.709	*0.023*
IL-10	0.130		1.139 (1.026–1.265)	3.450	*0.015*
IL-6	0.005		1.005 (0.999–1.011)	3.470	*0.082*
IL-1β	0.154		1.116 (0.713–1.906)	1.181	0.540

B: regression coefficient; OR: odds ratio; CI: confidence interval; Std. OR: odds ratio standardized for standard deviation of continuous predictors (e^B*SD^). Total patients n = 136 (control patients excluded); patients without catecholamine treatment (w/o Norepi.) n = 109 (on day 1).

**Table 3:** Results of univariate binary logistic regression analysis for bacterial infection d2–d7.

	B	OR (95% KI)	Std. OR	p value
Gender (male)	−0.165	0.848 (0.385–1.866)		0.682
Age	−0.009	0.992 (0.961–1.023)	0.897	0.592
NIHSSS	0.119	1.126 (1.075–1.180)	3.402	*<0.001*
Volume	0.005	1.005 (1.001–1.009)	1.620	*0.010*
Catechol. treat.	2.649	14.143 (5.55–36.05)		*<0.001*
Mechan. ventilation >24 h	3.339	28.20 (9.152–86.895)		*<0.001*
*Medical condition and premedication*				
Diabetes	−0.616	0.540 (0.188–1.548)		0.251
PrevCardioVasc	−0.668	0.513 (0.203–1.295)		0.158
β-AR blocker	−0.677	0.508 (0.210–1.231)		0.134
Smoking	−0.344	0.709 (0.262–1.920)		0.499
*Blood biomarkers at admission (d1)*				
Lymphocytes (absolute)	−0.123	0.885 (0.836–0.937)	0.307	*<0.001*
Lymphocytes <13%	1.852	6.375 (2.688–15.118)		*<0.001*
Noradrenalin	0.000	1.000 (1.000–1.000)	1.000	*0.005*
Normetanephrin	0.009	1.009 (1.001–1.016)	1.574	*0.019*
Noradrenalin (w/o Norepi.)	0.000	1.000 (1.000–1.000)	1.000	*0.455*
Normetanephrin (w/o Norepi.)	−0.005	0.995 (0.980–1.011)	1.033	*0.544*
ACTH	0.002	1.002 (0.998–1.006)	1.198	0.386
Cortisol	0.001	1.001 (0.999–1.003)	1.196	0.392
IL-10	0.065	1.067 (1.004–1.135)	1.826	*0.037*
IL-6	0.010	1.010 (1.002–1.017)	12.040	*0.014*
IL-1β	0.324	1.383 (0.838–2.280)	1.419	0.204
*Infection markers d2–d7 (all negative at d1)*				
CRP>5 mg/l	2.755	15.725 (2.060–120.021)		*0.008*
Temp.>38°C	1.617	5.036 (2.232–11.362)		*<0.001*
hsPCT (max.)	25.4	1.1E+11 (4.6E+6-2.5E+15)	1.73E+13	*<0.001*
hsPCT ≥0.1 ng/ml	3.871	48.0 (14.217–162.063)		*<0.001*

B: regression coefficient; OR: odds ratio; CI: confidence interval; Std. OR: odds ratio standardized for standard deviation for continuous predictors (e^B*SD^). Total patients n = 136 (control patients excluded); patients without catecholamine treatment (w/o Norepi.) n = 104 (during first week).

Multivariate binary regression analysis detected NIHSSS and age as independent predictors of lymphocytopenia<13%, but only NIHSSS had a significant p value <0.05 (**Table 4**). Mechanical ventilation >24 h and hsPCT values >0.1 ng/ml were found as independent predictors of bacterial infections (**Table 5** **)**. We performed an additional analysis for independent predictors of bacterial infections excluding the obviously correlated infection markers fever, CRP and hsPCT from the model (**Table 6**) and detected mechanical ventilation and lymphocytopenia on admission as independent predictors.

**Table 4:** Results of multivariate binary logistic regression analyses for independent predictors of lymphocytes <13% on day 1.

Lymphocytes <13%				
	Regr. coefficient	OR (95% CI)	Std. OR	p value
NIHSSS	0.141	1.151 (1.092–1.213)	4.267	<0.001
Age	0.038	1.099 (0.999–1.080)	1.581	0.053

Variables in the model: Age, NIHSSS, volume, smoking, catecholamine treatment d1, noradrenalin, cortisol; method: forward (likelihood ratio), p(in) = 0.05, p(out) = 0.10.

**Table 5:** Results of multivariate binary logistic regression analyses for independent predictors of bacterial infections during d2–d7.

Bacterial infection d2–d7			
	Regr. coefficient	OR (95% CI)	p value
Mechan. ventilation >24 h	2.931	18.744 (4.559–77.061)	<0.001
hsPCT >0.1 ng/ml	3.247	25.710 (6.555–100.831)	<0.001

Variables in the model: NIHSSS, volume, catecholamine treatment, noradrenalin, normetanephrin, temp. >38°C, CRP>5 mg/l, lymphocytes <13%, hsPCT>0.1, mechan. ventilation; method: forward (likelihood ration), p(in) = 0.05, p(out) = 0.10.

**Table 6:** Results of multivariate binary logistic regression analyses for independent predictors of bacterial infections during d2–d7 excluding obvious infection markers.

Bacterial infection d2–d7 (excluding the infection markers fever, CRP and hsPCT)			
	Regr. coefficient	OR (95% CI)	p value
Mechan. ventilation >24 h	3.120	22.638 (6.457–79.376)	<0.001
Lymphocytes <13% on d1	1.417	4.126 (1.430–11.907)	0.009

Variables in the model: NIHSSS, volume, catecholamine treatment, noradrenalin, normetanephrin, lymphocytes <13%, mechan. ventilation; method: forward (likelihood ration), p(in) = 0.05, p(out) = 0.10.

### Diagnostic Performance of Predictors for Lymphocytopenia and Bacterial Infections

**Figure 4:**
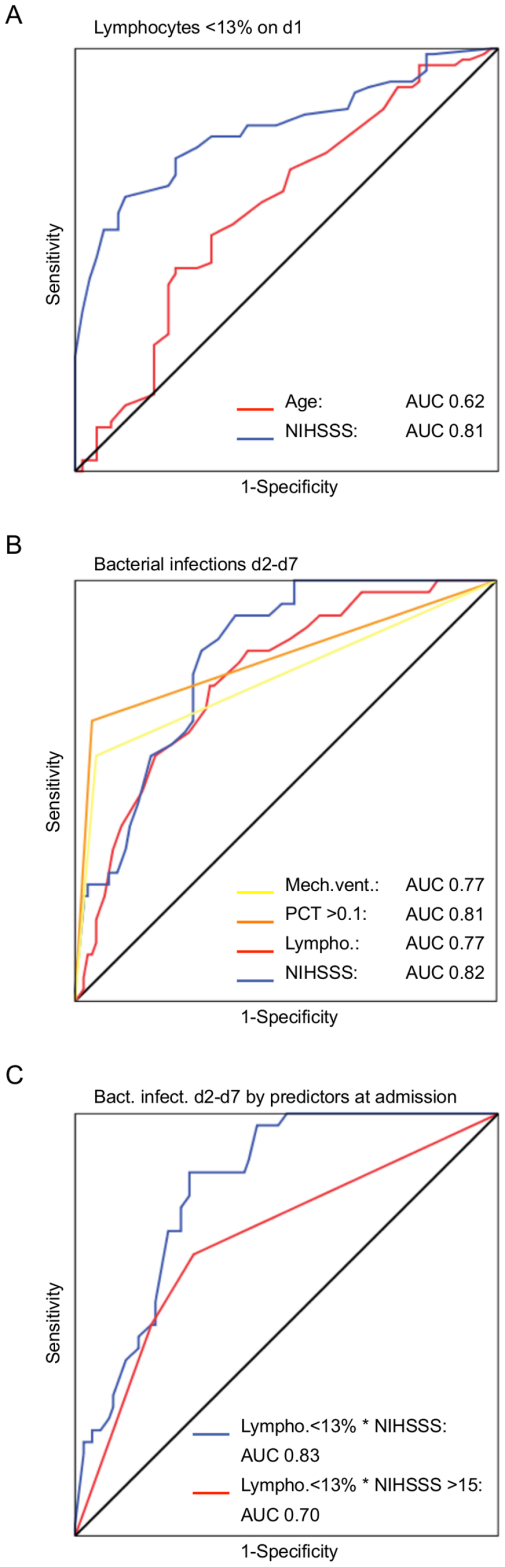
ROC curve analysis of independent predictors for lymphocytopenia and bacterial infections. (**A**) ROC curve performance of the independent variables age and NIHSSS for relative lymphocytopenia on admission (day1). (**B**) ROC curves of the independent predictors of bacterial infections as detected by multivariate regression analysis. NIHSSS and lymphocytes on admission (day 1) and mechanical ventilation and PCT during the first week. Lymphocytes were plotted as probabilities to avoid curve inversion by the negative correlation. (**C**) Analysis of ROC performance for the combined predictive variables available on admission. AUC: area under the curve.

We further performed receiver operating characteristic (ROC) curve analysis for the performance of the detected independent predictors as diagnostic criteria (**Fig. 4**). Analyzing the independent predictors of lymphocytopenia on admission, NIHSSS had an area under the curve (AUC) of 0.81, indicating a good discriminative power (**Fig. 4A**). Interestingly, analyzing predictors of bacterial infections during the first week revealed a similar ROC performance for NIHSSS on admission and hsPCT results during the first week as well as for lymphocytopenia on admission and mechanical ventilation during the first week (**Fig. 4B**). Using contingency table analysis for lymphocytopenia<13% we found a test sensitivity of 75% and specificity of 67% and for NIHSSS>15 a sensitivity of 65% and specificity of 76% (both on day 1) for detection of subsequent bacterial infections during the first week. We therefore performed ROC analysis of combined explanatory and independent variables [lymphocytes<13%×NIHSS] and [lymphocytes<13%×NIHSS>15] revealing an excellent ROC performance (AUC 0.83) (**Fig. 4C**). Combining both predictors resulted in a low test sensitivity (51%) compared to the individual predictors, however, particularly the test specificity of 85% and negative predictive value of 82% of the combined predictor were exceptionally good.

## Discussion

The major findings of our study are that (1) only large ischemic and hemorrhagic strokes lead to increased plasma levels of some catecholamine metabolites and cortisol. (2) Extensive lesions are associated with pronounced lymphocytopenia, increased cytokine secretion and a strong acute phase reaction. (3) Signs of bacterial infection including positive culture results or elevated hsPCT increase after cerebrovascular events. (4) Catecholamine and steroid levels were neither independent predictors of lymphocytopenia nor of bacterial infection. (5) Disease severity (NIHSSS) was the only significant independent predictor of lymphocytopenia. (6) Lymphocytopenia on admission was an independent predictor of subsequent bacterial infections.

The activation of the SNS and the HPA axis and the modulation of the peripheral immune system are both well-known features of stroke [9], [14], [20]–[22]. This is the largest study to date investigating a wide panel of hormonal stress mediators. It also studied the previously uncharacterized categories of ICH and TIA. We detected only a relatively minor increase of plasma catecholamine whereas other pathways such as the acute phase reaction or cytokine levels were profoundly activated in the same patients. ACTH and cortisol were already increased on the first day after admission after large ICH and ischemia. A stroke-induced immunosuppression syndrome after brain ischemia was previously characterized by the pathophysiological hallmarks lymphocytopenia, cellular and humoral immune dysfunction and susceptibility to bacterial infections [10], [11], [16]. The key features of this syndrome also presented with a similar pattern of immunomodulation in patients with extensive brain ischemia and hemorrhage. In contrast no significant immune changes were observed in TIA patients.

The current concept of post-stroke immunosuppression makes a causal link between the two phenomena hormonal stress response and immune dysfunction after stroke. Accordingly, stress hormones, particularly catecholamines, would be signaling from the injured brain to immune cells [8], [10], [11], [16]. By now, 11 clinical studies [12], [14], [19], [21], [23]–[29] have investigated aspects of post-stroke immunosuppression in ischemic stroke of which only 5 studies were prospectively designed. Only one trial detected a correlation between stress mediators and lymphocyte number [28]. No clinical study has distinctly identified catecholamines as independent predictors of lymphocyte dysfunction. Notably, neither the experimental landmark publication by Prass et al. [8] nor any other experimental study in the field of post-stroke immunity measured catecholamine levels after stroke. Pharmacological blockade of beta-adrenoreceptors partially prevented the immune changes after severe experimental stroke in Prass’ study^8^. However, sympathetic denervation did not affect splenic cellularity in another report [22]. In view of these limited and partially controversial findings the causal relationship between the stress response, immune dysfunction and infectious complications which has been advocated in numerous reviews of the subject appears doubtful[7], [9]–[11], [16], [30], [31]. Indeed, our detailed and extensive study challenges such a causal relationship as we did not detect an independent correlation of stress mediator plasma levels on one hand and systemic immune modulation and infectious complications on the other hand even in our large study cohort.

Lymphocytopenia is a hallmark phenomenon of immunodepression after both ischemic and hemorrhagic stroke. In the present study, lymphocytopenia was correlated with the severity of brain damage. The signals that may link stroke and lymphocytopenia are currently unknown. Ischemic and hemorrhagic brain lesions induced similar immune changes depending on lesion size. Conceivably, mediators directly released from the injured brain may trigger peripheral immune dysfunction. In this speculative concept, the amount of the released mediator and the extent of immune system modulation depend on the size of the lesion. Consistent with this concept, TIA patients with no parenchymal tissue damage did not have any changes in their immunological status in the present study. Since lesion volume was such a strong predictor of immune changes, we did not investigate individual brain areas previously suggested to be involved in immune control [32].

A clinically important issue is the lack of specific blood markers of infection. Components of the early phase reaction such as CRP, cytokines, and fever are frequently increased after stroke without other evidence of infection [19]. Multivariate logistic regression analysis in this study detected NIHSSS and lymphocytopenia on admission to be independent predictors of subsequent bacterial infections. Indeed, the very good negative predictive value of the discriminators NIHSSS>15 and lymphocytes<13% may serve as a diagnostic tool to avoid unnecessary antibiotic treatment [33], [34].

As a limitation of this study, the exploratory nature of the analysis may have limited the power of the statistical tests. Moreover, frequency and mode of testing for bacterial infections (e.g. cultures, chest x-ray) and antibiotic treatment regimens were not predefined but left to the treating physician’s discretion. Therefore, only positive microbiological bacterial culture results were used as the primary infection outcome parameter instead of combined infection scores using radiological or laboratory parameters. Third, we did not assess the direct interaction of sympathetic nerve fibers with immune cells, however, serum catecholamine are believed to reflect a systemic stress response.

In conclusion, our comprehensive analysis of the effects of acute cerebrovascular disorders on stress hormones, immune function and infections challenges the current concept of stress hormone mediated immunodepression after ischemic and hemorrhagic stroke. Stroke severity and lymphocyte number may serve as valuable predictors for early stratification of patients for antibiotic treatment.

## Supporting Information

Figure S1 **Dopamin plasma levels in patients with cerebrovascular diseases and control patients.** Plasma levels of dopamin were measured in control patients and in patients after TIA, ICH or stroke at the indicated time points. A significant reduction was observed only after small infarcts compared to TIA patients and controls at d1 after stroke. Data is presented as median and interquartile range. * indicates significant (p<0.05) difference of the indicated group compared to the control group; horizontal bar indicates significant difference between respective groups.
(TIF)
